# Supplementary material for: Identification of photoperiod-induced specific miRNAs in the adrenal glands of Sunite sheep (Ovis aries)
Source: Front Vet Sci. 2022 Jul 22;9:888207. doi: 10.3389/fvets.2022.888207 (PMC9354845; doi:10.3389/fvets.2022.888207)
Supplement: Supplementary file 1 [file Data_Sheet_1.ZIP › Supplementary file/miRDeep2 file.zip!/miRDeep2 file/Novel_Structure/SP42A2/Novel_624.pdf]

Provisional ID	:	NC_019459.2_7525
Score total	:	2.5
Score for star read(s)	:	-1.3
Score for read counts	:	0
Score for mfe	:	2.2
Score for randfold	:	1.6
Score for cons. seed	:	
Total read count	:	41
Mature read count	:	41
Loop read count	:	0
Star read count	:	0

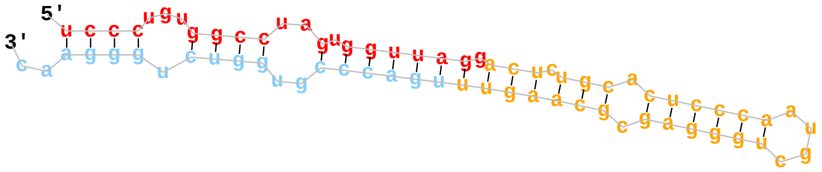

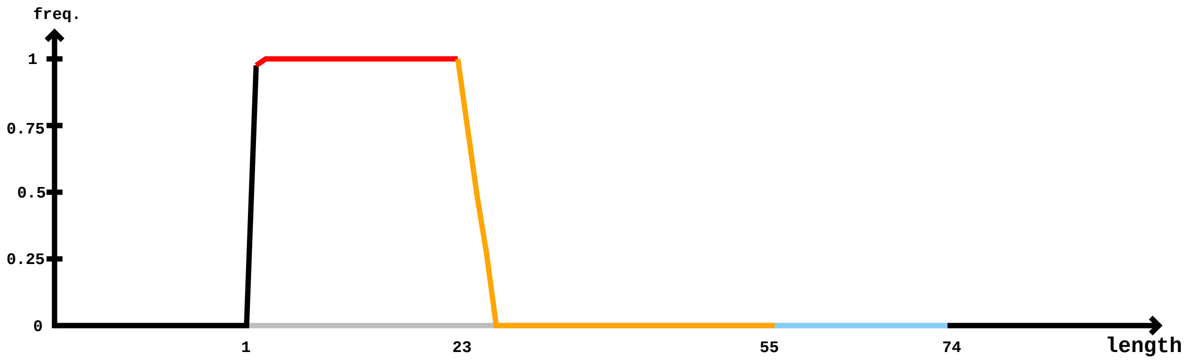

Mature **Star**

```
5'- ugaaagagguugugggacuucccuguggccuagugguuaggacucugcacucccaaugcugggagcgcaaguuugacccguggucugggaaccaagaucccacaugccgcaca   -3'    exp
    .....(.((((((((((((((((...((((..(.((((((.(((.(((.((((((....)))))).))))))))))))))..)))).))))....)).))))))).))).)...    reads     mm        sample
    ...................ucccuguggUcuagugguuagg........................................................................    15        1         seq
    ...................ucccuguggUcuagugguuagga.......................................................................    6         1         seq
    ...................ucccuguggUcuagugguuaggac......................................................................    9         1         seq
    ...................ucccuguggUcuagugguuaggacu.....................................................................    10        1         seq
    ....................cccuguggUcuagugguuaggacu.....................................................................    1         1         seq
```
